# Strengthening Primary Healthcare in Kosovo Requires Tailoring Primary, Secondary and Tertiary Prevention Interventions and Consideration of Mental Health

**DOI:** 10.3389/fpubh.2022.794309

**Published:** 2022-04-05

**Authors:** Katrina Ann Obas, Ariana Bytyci-Katanolli, Marek Kwiatkowski, Qamile Ramadani, Nicu Fota, Naim Jerliu, Shukrije Statovci, Jana Gerold, Manfred Zahorka, Nicole Probst-Hensch

**Affiliations:** ^1^Department of Epidemiology and Public Health, Swiss Tropical and Public Health Institute, Basel, Switzerland; ^2^University of Basel, Basel, Switzerland; ^3^Accessible Quality Healthcare Project, Prishtina, Kosovo; ^4^National Institute of Public Health, Prishtina, Kosovo; ^5^Medical Faculty, University of Prishtina, Prishtina, Kosovo; ^6^University Clinical Centre of Kosovo, Prishtina, Kosovo; ^7^Swiss Center for International Health, Swiss Tropical and Public Health Institute, Basel, Switzerland

**Keywords:** depressive symptoms, hypertension, diabetes, COPD, prevention, public health

## Abstract

**Objectives:**

Kosovo has the lowest life expectancy in the Balkans. Primary healthcare (PHC) plays an essential role in non-communicable disease (NCD) prevention. We described primary, secondary and tertiary prevention indicators in Kosovo and assessed their association with depressive symptoms.

**Methods:**

PHC users (*n* = 977) from the Kosovo NCD cohort baseline study were included. Depressive symptoms were assessed using the Depressive Anxiety Stress Scale-21. Cross-sectional associations between depressive symptoms and prevention indicators were quantified with mixed logistic regression models.

**Results:**

Poor nutrition (85%), physical inactivity (70%), obesity (53%), and smoking (21%) were common NCD risk factors. Many cases of hypertension (19%), diabetes (16%) and Chronic Obstructive Pulmonary Disease (COPD) (45%) remained undetected by a PHC professional. Uncontrolled hypertension (28%), diabetes (79%), and COPD (76%) were also common. Depressive symptoms were positively associated with physical inactivity (OR 1.02; 95% CI 1.00–1.05 per 1-point increase in DASS-21) and undetected COPD (OR 1.07; 95% CI 1.00–1.15), but inversely with undetected diabetes (OR 0.95; 95% CI 0.91–1.00).

**Conclusions:**

Continued attention and tailored modifications to primary, secondary and tertiary prevention in Kosovo are needed to narrow the Balkan health gap.

## Introduction

Over the last 30 years, there has been a discernible shift toward a greater proportion of the global disease burden (GBD) caused by non-communicable diseases (NCDs) (1). In 2019, NCDs accounted for 1,620,165,811 Disability Adjusted Life-Years (DALYs) or 64% of all DALYs globally, up from 43% in 1990 (2). Although the NCD burden in Kosovo is not well documented in part due to a health information system that is not yet fully functional (3), a heavy disease burden is evident from the considerably lower life expectancy (72.5 years) compared to neighboring countries such as Albania (78.6 years), Montenegro (76.9 years), North Macedonia (75.5 years), and Serbia (75.7 years) (4).

NCD management interventions are essential for achieving the SDG target of a one-third reduction in premature deaths from NCDs by 2030. The World Health Organization supports efforts toward achieving the 2030 Sustainable Development Goal 3 aimed at reducing NCD-related premature deaths by one-third by 2030. In fact, a Global Action Plan for the Prevention and Control of NCDs (5) was developed to help states reduce the burden of NCDs. Primary healthcare (PHC) plays an important role in NCD prevention and control (6). The Accessible Quality Healthcare (AQH) is a prominent project in Kosovo which is funded by the Swiss Agency for Development and Cooperation (SDC) and has been working with local stakeholders since 2016 to improve the quality of PHC in the public sector through a health system strengthening approach and with a focus on NCDs.

PHC has interventions at each stage of disease: Primary prevention aims to prevent the onset of disease through health promotion, secondary prevention aims to detect diseases early in an asymptomatic stage so that treatment can delay or block the occurrence of symptoms, and tertiary prevention attempts to deter adverse consequences of existing clinical disease (7, 8).

A rapid assessment of the PHC system in Kosovo conducted by the World Health Organisation (WHO) in 2019 (9) found that hospitalizations related to hypertension and diabetes decreased rapidly between 2012 and 2016, indicating major improvements in disease management in general (tertiary prevention). However, the life expectancy gap between Kosovo and its neighbors still exists in 2021. Equivalent data for chronic respiratory disease, and especially chronic obstructive pulmonary disease (COPD) is lacking. Identifying areas for improvement along the chain of care for common NCDs, i.e., in primary, secondary and tertiary prevention, can facilitate evidence-informed policymaking for PHC stakeholders in Kosovo and AQH project interventions.

A potentially important barrier to NCD prevention and control in Kosovo is poor mental health. as a post-conflict outcome. Depressive symptoms among PHC users were reported to be about 10% worldwide (10), while the prevalence of depressive symptoms reported in Kosovo far exceeds this, ranging from 30 to 67% (11–15). Depression has been linked to unhealthy behaviors such as smoking, physical inactivity, poor nutrition and alcohol consumption (16, 17). Depression has also been linked with uncontrolled hypertension (18–20) and poor glycemic control among diabetics (21) in other settings. Given the high prevalence of depressive symptoms reported in Kosovo, it is important to investigate its role in NCD management in the specific context.

**Figure 1:**
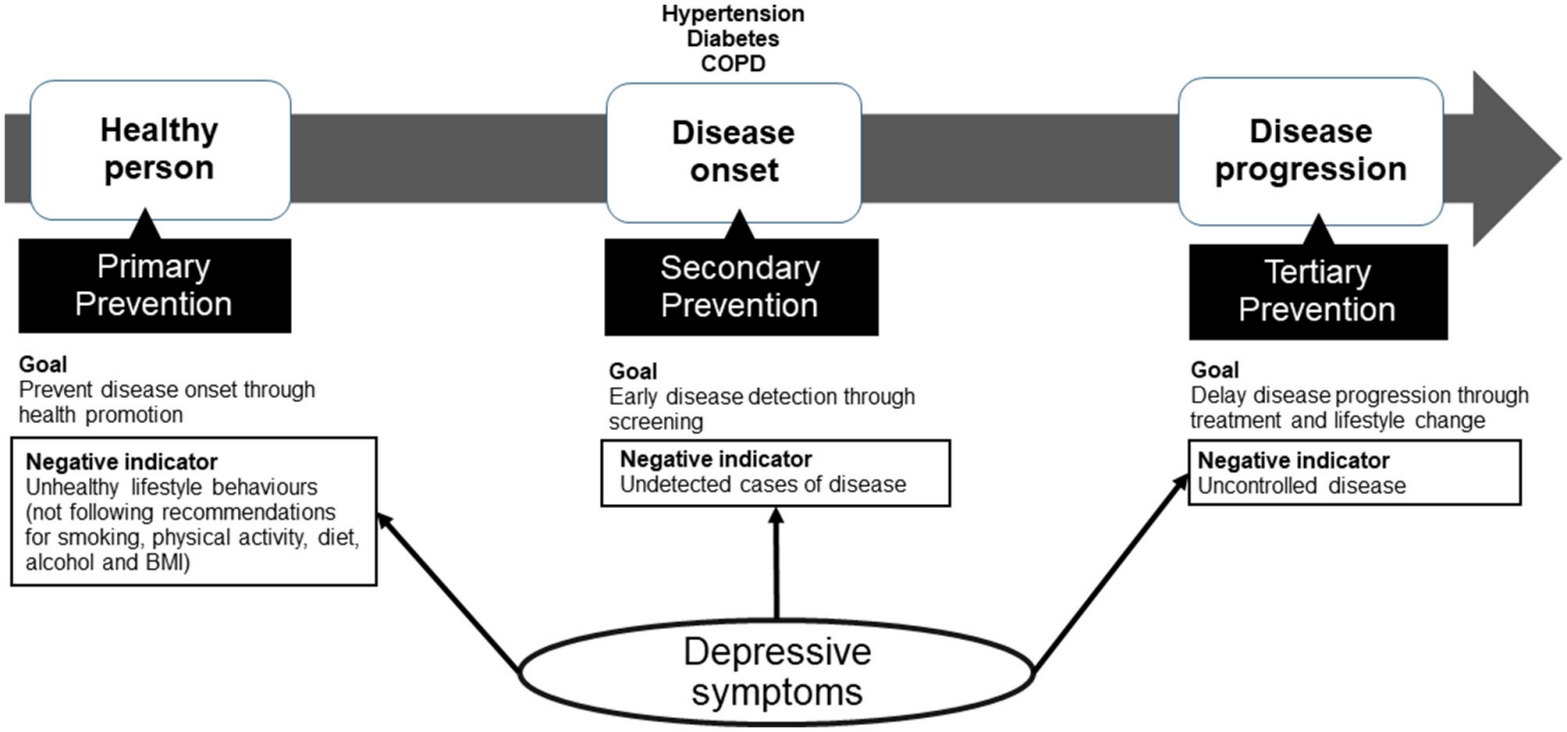
Conceptual framework. Primary care participants exist on a disease continuum, from a healthy person to disease onset, to disease progression. Primary healthcare aims to prevent people from moving forward along the continuum. Primary, secondary and tertiary prevention strategies target people at different stages of the disease continuum. To evaluate gaps in primary, secondary and tertiary prevention in Kosovo's PHC system, our study assesses negative indicators of each stage of prevention. Specifically, it describes the prevalence of lifestyle risk factors (targets if primary prevention), as well as undetected hypertension, diabetes and chronic obstructive pulmonary disease (COPD) (targets of secondary prevention) and uncontrolled hypertension, diabetes and COPD (targets of tertiary prevention) among Kosovo PHC users. It further assesses the association between depressive symptoms and these indicators to evaluate whether depressive symptoms act as a barrier to disease prevention.

Figure 1 depicts the study's conceptual framework. Primary care participants represent different stages of a disease continuum, from a healthy person to disease onset, to disease progression. Primary healthcare aims to prevent people from moving forward along the continuum. Primary, secondary and tertiary prevention strategies target people at different stages of the disease continuum. Our study assessed the distribution of negative cross-sectional indicators to identify gaps at the different prevention stages which may hinder NCD control in the Kosovo primary health care system. Specifically, it describes the prevalence of NCD risk factors (targets of primary prevention), as well as the prevalence of undetected hypertension, diabetes and chronic obstructive pulmonary disease (COPD) (targets of secondary prevention) and uncontrolled hypertension, diabetes and COPD (targets of tertiary prevention) among Kosovo public PHC users. It further assesses the association between depressive symptoms and these indicators to evaluate whether depressive symptoms act as a barrier to disease prevention and control.

## Materials and Methods

### Study Design

The current cross-sectional study uses baseline data of the KOSovo NCD COhort (KOSCO), which began in March 2019. Details of the study protocol are described elsewhere (22). In brief, the overarching goal of the KOSCO study was to contribute epidemiological evidence to the prevention and control of NCDs in the Kosovo public primary health care system as the basis for policy and decision-making. Initially, 1,011 consecutive PHC users aged 40 years and above were recruited from 12 PHC facilities in Kosovo. The data collected through interviews and health examinations included: socio-demographic characteristics, social and environmental factors, comorbidities, health system, lifestyle, psychological factors, and clinical attributes (blood pressure, height, weight, waist/hip/neck circumferences, peak expiratory flow and HbA1c measurements). Cohort data were collected annually in two phases, approximately 6 months apart, with an projected total follow-up time of 5 years. The current study is based on cross-sectional data from the baseline assessment.

### Setting

The study was conducted in Kosovo, which is situated in the middle of the Western Balkans and has a population of ~1.8 million. In Kosovo, the public PHC system is divided into three tiers: each municipality has one main family medicine centre (MFMC), several family medicine centres (FMC) and several family medicine ambulantas (FMA). MFMCs are the largest facilities at the highest level of PHC, which offer more services, employ more staff and have more medical equipment and therefore have a higher patient flow compared with the second-level FMCs and third-level FMAs. There is also the private PHC sector with only one tier consisting of private clinics.

Study sites include the MFMCs from the following 12 municipalities: Gračanica, Drenas, Skënderaj, Malishevë, Rahovec, Gjakovë, Junik, Fushë Kosovë, Vushtrri, Mitrovicë, Lipjan, and Obiliq.

### Participants

Recruitment and baesline data collection were conducted between March and November 2019. A total of 1,011 consecutive and consenting PHC users were included in the cohort. Ethical approvals were obtained from the Ethics Committee Northwest and Central Switzerland (Ref. 2018-00994) and the Kosovo Doctors Chamber (Ref. 11/2019).

Participants were included in the cohort if they were aged 40 years or older and consulted healthcare services irrespective of the reason on the day of recruitment. Participants were excluded from the cohort if they had a terminal illness, were not able to understand or respond to screening questions, did not live in one of the 12 study municipalities, or lived abroad for more than 6 months of the year.

Baselien participants who had complete data on confounders (age, sex, work status, highest level of education achieved, living in a rural or urban setting, and ethnicity), smoking status, physical activity, nutrition, alcohol, height, weight, blood pressure, glycated hemoglobin (HbA1c), peak expiratory flow (PEF), depressive symptoms score, and status of hypertension, diabetes and COPD diagnoses were included in the current study (*n* = 977). We excluded 34 participants due to incomplete data.

### Variables and Data Sources

#### NCD Risk Factors

Participants answered questions during an in-person interview with a trained study nurse regarding lifestyle and mental health symptoms. Height (in meters) and weight (in kilograms) were measured, body mass index (BMI) was derived (weight/height^∧^2). The following indicator variables for NCD risk factors were defined as follows:

- smoking status (current smoker).
- physical inactivity (<150 min of moderate-intensity physical activity per week, <75 min of vigorous-intensity physical activity per week, and less than an equivalent combination of moderate-intensity and vigorous-intensity activity).
- Poor nutrition (<5 fruit and/or vegetable portions per day).
- Alcohol consumption (any alcohol consumed in the last 30 days).
- Obesity (BMI ≥ 30).

A lifestyle index was equally derived by taking the sum of the indicators above, where one point was given for each criterion met.

#### Undetected and Uncontrolled Disease

Participants answered questions about physician-diagnosed hypertension, diabetes and COPD. Blood pressure, HbA1c and PEF were measured at the end of the interview. Systolic and diastolic blood pressures (in mmHg) were measured three times, at least 3 min apart, after sitting quietly for about 10 min, using an M3 model Omron blood pressure monitor (Omron Healthcare, Switzerland). The research nurses placed the blood pressure cuff 2 cm above the elbow on the bare left upper arm (in the case of arteriovenous fistula, radiotherapy or removal of lymph nodes in the armpit of the left arm, the right arm was used) of the seated participant and elevated the arm on the table to the level of the fourth intercostal space. The non-invasive (finger-prick blood sample) HbA1c test was performed by the MFMC staffed laboratory technician who received training from the supplier on how to use the SUPER ID clinchem device (Dr. Müller Gerätebau GmbH, Germany). In the absence of sufficient funds for the conduct of spirometry to assess irreversible obstruction to air flow for COPD assessment, PEF (L/min) was measured 3 times with a 30-second pause between attempts, using the OMRON Peak Flow Meter PFM20 (Omron Healthcare, Switzerland). PEF predicted (%) was calculated as the ratio of the estimated (measured) PEF to the expected PEF. The expected PEF values were derived based on age, gender and height using the regression equation developed by Hankinson et al. (23). From these data sources, indicator variables of undetected and uncontrolled hypertension, diabetes and COPD variables were defined as follows:

- Undetected hypertension: no self-reported physician diagnosis of hypertension as well as systolic blood pressure ≥140 mmHg or diastolic blood pressure ≥90 mmHg.
- Undetected diabetes: no self-reported physician diagnosis of diabetes and HbA1c ≥ 6.5%.
- Undetected COPD: no self-reported physician diagnosis of COPD as well as PEF <80% Predicted (24) with breathlessness for 6 months or longer or cough for at least 3 months.
- Uncontrolled hypertension: self-reported physician diagnosis of hypertension as well as systolic blood pressure ≥140 mmHg or diastolic blood pressure ≥90 mmHg.
- Uncontrolled diabetes: self-reported physician diagnosis of diabetes and HbA1c ≥ 6.5%.
- Uncontrolled COPD: self-reported physician diagnosis of COPD and PEF <80% Predicted.

Undetected and uncontrolled diseases were further stratified by stage of the disease:

- Hypertension: Stage 1 is systolic blood pressure ≥140 mmHg or diastolic blood pressure ≥90 mmHg and stage 2 is systolic blood pressure ≥160 mmHg or diastolic blood pressure ≥100, according to the international society of hypertension (25).
- Diabetes: Level 1 is an HbA1c 6.5–7.4%, level 2 is an HbA1c 7.5–9.0%, level 3 is an HbA1c 9.1–11%, and level 4 is an HbA1c > 11% (26).
- COPD: level 1 is a PEF 50–79% Predicted, level 2 is a PEF <50% predicted.

#### Depressive Symptoms

Depressive symptoms were measured using the Depressive Anxiety Stress Scale (DASS-21) (27), a 21-item questionnaire consisting of subscales for depressive, anxiety and stress symptoms, each containing seven items scored on a 4-point Likert scale ranging from 0 (did not apply to me at all) to 3 (applied to me very much). The sum of scores from the depressive symptoms subscale was then multiplied by 2. The depressive symptoms scores range from 0 to 42.

#### Statistical Analyses

Sociodemographic factors among PHC users were presented as frequency and percentages for categorical variables and as the median and interquartile range for non-normal distributed continuous variables.

The prevalence of NCD risk factors (smoking, physical inactivity, poor nutrition, alcohol consumption, obesity and lifestyle index) among PHC users were described as frequencies and percentages for the total study population and were stratified by sex (male, female) and highest education level attained (primary school, secondary school, university). The prevalences of undetected and uncontrolled hypertension, diabetes and COPD among PHC users were presented as frequency and percentages of the relevant subsample, and also stratified by sex and highest education level attained. For the outcome of undetected disease, participants were included in the subsample if they had either a diagnosis for the disease or pathological clinical findings for that disease (systolic blood pressure ≥ 140 mmHg or diastolic blood pressure ≥ 90 mmHg for hypertension; HbA1c ≥ 6.5% for diabetes; PEF <80% Predicted with breathlessness for 6 months or longer or cough for at least 3 months for COPD). For the outcome of uncontrolled disease, only those with a self-reported doctor's diagnosis of the disease were included in the subsample.

The adjusted cross-sectional associations between depressive symptoms as a continuous predictor variable and outcomes of smoking, physical inactivity, alcohol consumption, poor nutrition, and obesity, undetected hypertension, diabetes and COPD as well with uncontrolled hypertension and diabetes were quantified using mixed logistic regression models, while mixed ordinal logistic regression was used for the association between depressive symptoms and lifestyle index. Municipality (Gračanica, Drenas, Skënderaj, Malishevë, Rahovec, Gjakovë, Junik, Fushë Kosovë, Vushtrri, Mitrovicë, Lipjan, and Obiliq) was included as a random effect in all models. We selected potential confounders for inclusion in these models based on prior knowledge: age (years), sex (male, female), work status (currently working, house person, retired or disabled, unemployed), highest level of education achieved (primary school, secondary school, university), living in a rural or urban setting (rural, urban), and ethnicity (Albanian, Serbian, Roma or Ashkali or Egyptian or Other). Due to few observed cases of alcohol consumption in the last 30 days, the model of the association between depressive symptoms and alcohol was reduced to include only age, sex and ethnicity as confounders. Due to even fewer cases of uncontrolled COPD, a regression model for the association between depression and uncontrolled COPD was not interpretable. The same subsamples of undetected and uncontrolled disease apply to the regression models as the descriptive outcomes. The same methods were applied with depressive symptoms as a binary predictor variable and are available (moderate to very severe depressive symptoms equate to a DASS-21 depressive symptoms score ≥14) in the supplementary data (Supplementary Table 1).

Analyses were performed with Stata statistical software, release 16.

## Results

### Sociodemographic Characteristics

We included 977 participants from KOSCO in this study. The participant characteristics are described in Table 1. There were more women than men in the study, and most had attained primary school education or less. Most participants were not working. The majority of participants identified as ethnic-Albanian and living in rural settings.

**Table 1:** Participant characteristics (Kosovo Non-Communicable Disease Cohort, Kosovo, 2019).

**Sociodemographic factors**	**All participants (*n* = 977)**
Age, median (IQR)	60 (53–67)
**Sex, frequency (%)**	
Male	402 (41.2)
Female	575 (58.8)
**Education, frequency (%)**	
Primary school or less	618 (63.3)
Secondary school	300 (30.7)
University/College	59 (6.0)
**Work status, frequency (%)**	
Currently working	162 (16.6)
House person	467 (47.8)
Retired or disabled	314 (32.1)
Unemployed	34 (3.5)
**Residence, frequency (%)**	
Rural	549 (56.2)
Urban	428 (43.8)
**Municipality, frequency (%)**	
Drenas	96 (9.8)
Fushe Kosova	109 (11.2)
Gjakova	72 (7.4)
Gracanica	52 (5.3)
Junik	21 (2.2)
Lipjan	171 (17.5)
Malisheva	77 (7.9)
Mitrovica	81 (8.3)
Obiliq	70 (7.2)
Rahovec	77 (7.9)
Skenderaj	93 (9.5)
Vushtrri	58 (5.9)
**Ethnicity, frequency (%)**	
Albanian	890 (91.1)
Serbian	48 (4.9)
Roma, Ashkali, Egyptian, Other	39 (4.0)
**Clinical measurements**	
Blood pressure (mmHg),	
Systolic, median (IQR)	133 (123–146)
Diastolic, median (IQR)	86 (80–93)
HbA1c (%), median (IQR)	6.5 (5.7–7.7)
PEF (L/min), median (IQR)	260 (187–350)
BMI, median (IQR)	30.3 (27.4–34.1)
**Disease**	
Diagnosed hypertension, freq (%)	605 (61.9)
Diagnosed Diabetes, freq (%)	506 (51.8)
Diagnosed COPD, freq (%)	59 (6.0)
Depressive symptoms score (median, IQR)	2 (0–6)
**Depressive symptoms severity (freq, %)**	
Normal (DASS depression score 0–9)	792 (81.1)
Mild (DASS depression score 10–13)	66 (6.8)
Moderate (DASS depression score 14–20)	84 (8.6)
Severe (DASS depression score 21–27)	17 (1.7)
Very severe (DASS depression score 28–42)	18 (1.8)

*IQR, Interquartile range; mmHg, millimeters of mercury; HbA1c, glycated hemoglobin; PEF, Peak Expiratory Flow; BMI, body mass index; COPD, chronic obstructive pulmonary disease; DASS, Depression Anxiety Stress Scale*.

### Primary Prevention

Table 2 describes the prevalence of NCD risk factors in the PHC users. More than 40% of participants reported 3 or more unhealthy lifestyle factors. Prevalence was highest for poor nutrition, with 85.1% reporting insufficient fruit and vegetable consumption, followed by 70.3% reporting physical inactivity, and 52.7% being obese. There existed important gender differences for smoking, alcohol consumption and obesity. Obesity and multiple unhealthy lifestyles were common among participants of low socioeconomic status, while higher socioeconomic status privileged smoking.

**Table 2:** Prevalence of non-communicable disease risk factors, stratified by sex and highest level of education attained (Kosovo Non-Communicable Disease Cohort, Kosovo, 2019).

**Risk factor**	**All participants (*n* = 977)**	**Sex**			**Highest level of education attained**			
		**Male (*n* = 402)**	**Female (*n* = 575)**	***p*-value**	**Primary (*n* = 618)**	**Secondary (*n* = 300)**	**University (*n* = 59)**	***p*-value**
Current smoker	201 (20.6)	110 (27.4)	91 (15.8)	<0.001*^a^	106 (17.2)	77 (25.7)	18 (30.5)	0.002*^a^
Physical inactivity	687 (70.3)	250 (62.2)	437 (76.0)	<0.001*^a^	482 (78.0)	165 (55.0)	40 (67.8)	<0.001*^a^
Poor nutrition	831 (85.1)	340 (84.6)	491 (85.4)	0.725 ^a^	537 (86.9)	246 (82.0)	48 (81.4)	0.106^a^
Alcohol consumption	44 (4.5)	43 (10.7)	1 (0.2)	<0.001*^a^	14 (2.3)	26 (8.7)	4 (6.8)	<0.001*^a^
Obesity	515 (52.7)	151 (37.6)	364 (63.3)	<0.001*^a^	381 (61.7)	111 (37.0)	23 (39.0)	<0.001*^a^
Lifestyle index ^b^				<0.001*^c^				<0.001*^c^
0	16 (1.6)	13 (3.2)	3 (0.5)		2 (0.3)	11 (3.7)	3 (5.1)	
1	155 (15.9)	81 (20.2)	74 (12.9)		69 (11.2)	77 (25.7)	9 (15.3)	
2	379 (38.8)	156 (38.8)	223 (38.8)		240 (38.8)	115 (38.3)	24 (40.7)	
3	348 (35.6)	112 (27.9)	236 (41.0)		259 (41.9)	72 (24.0)	17 (28.8)	
4	74 (7.6)	35 (8.7)	39 (6.8)		46 (7.4)	23 (7.7)	5 (8.5)	
5	5 (0.5)	5 (1.2)	0 (0.0)		2 (0.3)	2 (0.7)	1 (1.7)	

^a^Chi^2^ test. ^b^Lifesyle index is the sum of indicators for current smoker, physical inactivity, poor nutrition, alcohol consumption and obesity. ^c^Kruskal-Wallis test. *p < 0.05.

### Secondary and Tertiary Prevention

Table 3 describes the prevalence of undetected (secondary prevention) and uncontrolled (tertiary prevention) diseases. Many cases of hypertension (19%), diabetes (16%) and COPD (45%) remained undetected by a healthcare professional in PHC facilities. Uncontrolled disease was also very common in diabetes patients (79%) and COPD patients (76%). Most undetected cases of hypertension and diabetes were within the lower stages of the disease, but at higher stages of disease for uncontrolled hypertension and diabetes. There were important sex differences in the detection and control of COPD. Highly educated people tended to have higher undetected hypertension and people with less education had a higher prevalence of uncontrolled COPD.

**Table 3:** Prevalence of undetected and uncontrolled hypertension, diabetes and COPD, also disaggregated by disease severity and stratified by sex and educational level (Kosovo Non-Communicable Disease Cohort, Kosovo, 2019).

**Category**	**All participants of subsample**	**Sex**			**Highest level of education attained**			
		**Male**	**Female**	***P*-value^**a**^**	**Primary**	**Secondary**	**University**	***P*-value^**a**^**
Undetected hypertension (*n* = 743) SBP 140–159 or DBP 90–99 SBP ≥ 160 or DBP ≥ 100	138 (18.6) 111 (14.9) 27 (3.6)	70 (24.0)	68 (15.1)	0.002*	75 (15.3)	51 (23.6)	12 (31.6)	0.004*
Undetected diabetes (*n* = 601) Hba1c 6.5–7.4% Hba1c 7.5–9.0% Hba1c 9.1–11.0% Hba1c >11.0%	95 (15.8) 67 (11.2) 22 (3.7) 4 (0.7) 2 (0.3)	36 (14.7)	59 (16.7)	0.478	62 (16.3)	27 (14.5)	6 (17.7)	0.826
Undetected COPD (*n* = 108) PEF predicted 50–70% PEF predicted <50%	49 (45.4) 25 (23.2) 24 (22.2)	13 (36.1)	36 (50.0)	0.172	35 (46.7)	13 (43.3)	1 (33.3)	0.870
Uncontrolled hypertension (*n* = 605) SBP 140–159 or DBP 90-99 SBP ≥160 or DBP ≥100	171 (28.3) 73 (12.1) 98 (16.2)	74 (33.3)	97 (25.3)	0.350	114 (27.5)	49 (29.7)	8 (30.8)	0.837
Uncontrolled diabetes (*n* = 506) Hba1c 6.5–7.4% Hba1c 7.5–9.0% Hba1c 9.1–11.0% Hba1c >11.0%	400 (79.1) 150 (29.6) 146 (28.9) 86 (17.0) 18 (3.6)	170 (80.6)	230 (78.0)	0.478	258 (80.9)	121 (76.1)	21 (75.0)	0.416
Uncontrolled COPD (*n* = 59) PEF predicted 50–70% PEF predicted <50%	45 (76.3) 29 (49.2) 16 (27.1)	15 (65.2)	30 (83.3)	0.111	32 (80.0)	12 (70.6)	1 (50.0)	0.503

^a^Chi^2^ test, *p < 0.05. SBP, systolic blood pressure in mmHg; DBP, diastolic blood pressure in mmHg; HbA1c, glycated hemoglobin; PEF, peak expiratory flow; COPD, chronic obstructive pulmonary disease. Subsamples for undetected hypertension, diabetes and COPD included all participants with a self-reported physician diagnosis or pathological findings for the given disease (systolic blood pressure ≥ 140 mmHg or diastolic blood pressure ≥ 90 mmHg for hypertension; HbA1c ≥ 6.5% for diabetes; PEF <80% Predicted with breathlessness for 6 months or longer or cough for at least 3 months for COPD). Subsamples for uncontrolled disease included all participants with a self-reported physician diagnosis for the given disease. The vertical red line indicates the limit of the odds ratio of one. *p < 0.05.

### Association Between Depressive Symptoms Score and Primary, Secondary and Tertiary Prevention Indicators

**Figure 2:**
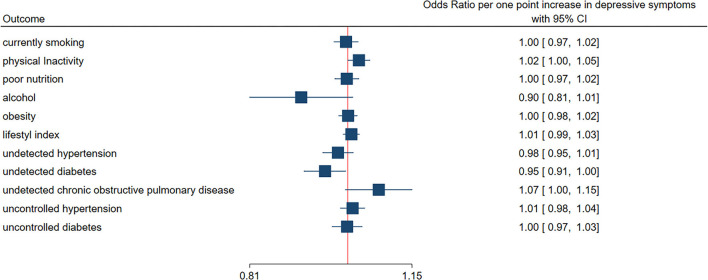
Association between depressive symptoms (continuous score) and primary, secondary and tertiary prevention negative indicators. Squares indicate the odds ratio per one-point increase in depressive symptoms. Lines indicate the 95% confidence interval. Depressive symptoms were assessed using the Depression, Anxiety, Stress Scale-21. Mixed ordinal logistic regression models quantified the association between depressive symptoms and lifestyle index. The associations between depressive symptoms and all other outcomes were quantified with mixed logistic regression models. All models included municipality as a random effect and were adjusted for age, sex, work status, education level, living in a rural or urban setting, and ethnicity with exception of alcohol, which was reduced to adjustment for only age, sex and ethnicity.

Figure 2 shows the adjusted associations between depressive symptoms score and NCD risk factors (smoking, physical, inactivity, poor nutrition, alcohol consumption, obesity, and unhealthy lifestyle index), undetected and uncontrolled hypertension, diabetes and COPD. Per one-point increase in depressive symptoms, we found the following trends: increase in odds of physical inactivity (OR 1.02; 95% CI 1.00–1.05), a decrease in odds for undetected diabetes (OR 0.95; 95% CI 0.91–1.00), and an increase in odds for undetected COPD (OR 1.07; 95% CI 1.00–1.15). The coefficients represented in Figure 2 are also available in table format available in the supplementary data (Supplementary Table 2).

## Discussion

The results of this study in Kosovo indicate that the need for improving NCD prevention and control in PHC remains high along the chain of care for disease prevention. NCD risk factors were common among PHC users. Many cases of hypertension, diabetes and possibly COPD remained undetected and also poorly controlled after their diagnosis. Depressive symptoms occurring at a high prevalence and being associated in particular with low levels of physical activity are an important control target and a potential barrier to the control or other NCDs.

### Primary Prevention

#### Smoking

In a national STEPS population-based survey (*n* = 6,117) conducted in 2010, it was found that 28.4% were smokers, where the prevalence was nearly double among men compared to women. The age group with the highest prevalence was between 35 and 44 years (28). We found a lower prevalence of smokers in our sample of PHC users aged 40 years and older in 2019. The finding that smoking was more prevalent among those with higher education level was surprising, but may indicate socioeconomic mechanisms, as a symbol of status. Our findings may point toward success in smoking cessation among adult smokers, but they may also reflect the generally lower smoking rates in older persons. Given the increasing concern about smoking among younger people in Kosovo (29) and a general concerning increase in popularity of electronic cigarettes (vaping) among adolescents worldwide (30), careful planning of interventions to prevent the onset of smoking at an early age must continue to avoid a future health burden due to tobacco in the coming decades.

#### Physical Inactivity

Our findings point toward very low adherence to WHO recommended guidelines for physical activity. A study comparing elderly populations in European countries found that Kosovo performed the worst among 28 countries (31). One national survey in Kosovo conducted in 2015 (32) among people aged 65 and older found that only 14.3% were practicing regular physical activity, where males (20.2%) reported regular physical activity more often than women (9.2%). Our study indicates that nearly 30% of PHC users aged 40 years and older are physically active. Although our sample is younger than the 2015 survey, it is still promising that progress is being made toward improving physical activity. Nevertheless, 70% of PHC users are still physically inactive and thus interventions promoting regular exercise should remain in the focus of PHC interventions. Furthermore, additional research needs to identify personal and structural barriers to physical acitivity in the specific context of Kosovo.

Depression has been associated with physical inactivity (16) and our study supports these findings in the Kosovo setting. This relationship suggests that depression may play a role as a barrier to interventions aimed at physical activity. Therefore, adequately treating depressive symptoms may improve physical activity interventions in itself and thereby propagating a positive feedback loop as physical activity interventions have a beneficial effect on depressive symptoms (33).

#### Poor Nutrition

We found that poor nutrition was very common (85%), meaning that most participants did not consume at least 5 servings of fruit and/or vegetables per day. There were no differences between sex and education level.

#### Alcohol Consumption

We found a very low prevalence of alcohol consumers in the last 30 days. These findings were unsurprising given that our sample includes older adults who may be more inclined toward traditional practices in a country that has a Muslim majority. Yet, the fact that both, alcohol consumption and smoking were more prevalent among more educated participants suggests that adults in Kosovo are adopting a Western European lifestyle. A shift toward unhealthy lifestyles that parallels the economic development of the country should be prevented at all costs.

#### Obesity

Over 50% of PHC users were obese and the prevalence was higher among people with lower levels of education. More women were obese compared to men. A previous small study (*n* = 423) conducted in two Kosovar communities in 2010 had only 30% obesity (34). Interventions in PHC targeting obesity should pay special attention to tailor to women and those of lower socioeconomic status.

#### Lifestyle Index

Women and those with lower education levels tended to have more unhealthy lifestyles overall compared to men and higher education levels respectively. There was no association between depressive symptoms and the number of unhealthy lifestyle factors after adjusting for confounders. This was unexpected given that depression has been associated with a cluster of unhealthy lifestyles (16). The results of this study point to physical inactivity as a central aspect of an unhealthy lifestyle in persons with signs of depression.

### Secondary Prevention

We found that nearly one in five PHC users with hypertension were unaware of it, nearly one in six with diabetes were not aware of it and nearly half with potential COPD were not aware of it. The diagnostic gap is scarcely studied in Kosovo. One study conducted in 2006 in adults 18 years and older (*n* = 423) found that over 1 in 3 people with pathological fasting glucose were not diagnosed with diabetes (34).

All of our participants received medical care for various reasons on the day of baseline data collection. Since 2019, the AQH project supports PHC with the implementation of WHO Package of Essential Non-Communicable Disease (PEN) Protocols (35) in five municipalities of Kosovo (Fushe Kosovo, Mitrovica, Malisheva, Gjakova and Vushtrri). The PEN protocol is used for the assessment and management of cardiovascular risk using hypertension, diabetes mellitus and tobacco use as entry points. It outlines screening recommendations for hypertension and diabetes in target groups through blood pressure and blood glucose measurement, which include people aged 40 years and over. Our findings highlight the importance of continuing and adapting the AQH efforts in implementing the PEN protocol and support its expansion to other municipalities and scaling up.

We found a high prevalence of potentially undetected COPD. In the absence of post-bronchodilation spirometry, though, the differentiation between COPD and/or asthma remains imperfect. But the results point to the importance of airflow obstruction. Although PEN protocols are available from the WHO for COPD, they were not yet implemented in Kosovo by AQH at the time of writing this paper. The MFMC directors requested that the study protocol include lung function testing for assessing respiratory disease. The stakeholder's clinical observations indicated that respiratory disease was a concern in their communities, and this study supports these observations. Further research on COPD with spirometric testing and considering also environmental factors is warranted. In Kosovo, Coal remains a common method to heat homes in the winter at a lower cost (36). The indoor air pollution from coal may be an important contributor to exacerbated respiratory disease.

Depressive symptoms were associated with lower odds of undetected diabetes in our adjusted models. This can be explained through increased healthcare utilization among people with depression (37). Underlying diabetes may be more likely to be diagnosed in people with depression since they are assessed and screened more often by a healthcare professional. A longitudinal study assessing healthcare utilization as a mediator of the association is warranted.

### Tertiary Prevention

Uncontrolled disease was very common among PHC users. Over a quarter of people with diagnosed hypertension still had high blood pressure. Even more troubling was that the majority, over three in every four PHC users with diagnosed diabetes or what we considered as potential COPD, had their disease uncontrolled. Men had more difficulty controlling their blood pressure and women had more trouble controlling their lung function, while no marked differences in sex were observed for diabetes control. There were no marked differences for control of hypertension and diabetes by education level, but less-educated participants were more likely to have uncontrolled COPD, possibly due to a combination of less access to care and poorer indoor environments, favoring disease progression.

### Strengths and Limitations

Our study comprehensively assesses the chain of care in the PHC system to identify specific areas for improvement. Given the current limited epidemiological data situation in the country, our findings provide evidence for stakeholders and decision-makers. Our study sample of PHC patients aged 40 years or more is not representative of the general population of Kosovo. However, the alarming prevalences of unhealthy lifestyle behaviors and poor detection and control of NCDs in our sample suggest that the current society-wide NCD prevention strategies are in urgent need of strengthening. While the prevalence estimates for unhealthy lifestyles might be an overestimation of the prevalence in the general population, given that we assessed PHC users, the fact that even in PHC users we observe a high rate of underdiagnosis and poor disease control could point to even higher rates in the general population with groups of people having very poor access to care.

Our findings brought to light the urgent need for further research on respiratory disease in Kosovo. We found that nearly half of people with problematic lung function were never diagnosed with COPD. Yet, peak flow measurements have limitations concerning detecting irreversible obstruction to airflow and cannot differentiate between COPD and asthma with high accuracy. It is foreseen for future cohort follow-ups to include pre- and post-bronchodilation spirometry as part of the health examinations.

Our study may be subject to response bias for the topics of alcohol and depression. In Kosovo, it is not generally well viewed to drink alcohol, especially among older adults and therefore participants may not have answered truthfully about alcohol intake. As most areas in the world, mental health is still a stigmatized topic in the country and similarly, participants may not have responded truthfully about their depressive symptoms. The prevalence of depression may therefore be an underestimation, which is particulary worrisome in the light of the recent COVID-19 pandemic, which may have increased the prevalence further. Although questions on alcohol were worded as per the WHO STEPS survey and we did not feel they needed to be changed, we decided to preface DASS questions with an introductory statement to frame the questions about wellbeing, which is a more socially acceptable topic.

The study is cross-sectional in nature and does not allow differentiating cause and effect in the association between depression and NCD control. The follow-up of the cohort will provide an opportunity in the future to address these associations longitudinally.

## Conclusion

An unhealthy lifestyle, undetected and uncontrolled hypertension, diabetes and possibly COPD are common in Kosovo, contributing to the heavy disease burden in the country. Disease prevention in Kosovo is improving, but still needs continued attention and tailored modifications to primary, secondary and tertiary prevention to narrow the health gap between Kosovo and other Balkan countries.

## Data Availability Statement

The raw data supporting the conclusions of this article will be made available by the corresponding author upon reasonable request.

## Ethics Statement

The study involving human participants were reviewed and approved by Ethics Committee Northwest and Central Switzerland (Ref. 2018-00994) and Kosovo Doctors Chamber (Ref. 11/2019). The patients/participants provided their written informed consent to participate in this study.

## Author Contributions

KO co-developed and implemented the study protocol, coordinated and supervised data collection, carried out the data analysis, interpreted results, and wrote the manuscript. NJ contributed to study objectives related to non-communicable diseases in Kosovo. SS contributed to study objectives related to mental health in Kosovo. MK supervised data analysis. MZ, QR, AB-K, and JG contributed to the study objectives related to the evaluation of health service provision and to the integration of the study protocol within the AQH framework. NP-H developed the KOSCO cohort concept, study objectives and protocol, directed the implementation, data analysis, and result interpretation. All authors have read and approved the final protocol.

## Funding

This study was funded by the Swiss Agency for Development and Cooperation.

## Conflict of Interest

The authors declare that the research was conducted in the absence of any commercial or financial relationships that could be construed as a potential conflict of interest.

## Publisher's Note

All claims expressed in this article are solely those of the authors and do not necessarily represent those of their affiliated organizations, or those of the publisher, the editors and the reviewers. Any product that may be evaluated in this article, or claim that may be made by its manufacturer, is not guaranteed or endorsed by the publisher.
