# Supplementary material for: Strengthening Primary Healthcare in Kosovo Requires Tailoring Primary, Secondary and Tertiary Prevention Interventions and Consideration of Mental Health
Source: Front Public Health. 2022 Apr 5;10:794309. doi: 10.3389/fpubh.2022.794309 (PMC9037373; doi:10.3389/fpubh.2022.794309)
Supplement: Supplementary file 1 [file Table_1.DOCX]

**Table S1.** Association between depressive symptoms (DASS-21 depressive symptoms score ≥14) and unhealthy lifestyle behaviours (smoking, physical, inactivity, poor nutrition, alcohol consumption, obesity, and unhealthy lifestyle index), undetected and uncontrolled hypertension, diabetes and COPD (Kosovo Non-Communicable Disease Cohort, Kosovo, 2019).

|  | Adjusted association with depression symptoms as a binary score (DASS-21 depressive symptoms score ≥14) | | |
| --- | --- | --- | --- |
| Outcome | Odds Ratio | 95 % confidence interval | |
| Currently smoking (n=977) | 0.85 | 0.49 | 1.49 |
| Physical inactivity (n=977) | 1.40 | 0.86 | 2.29 |
| Poor nutrition (n=977) | 0.83 | 0.47 | 1.44 |
| Alcohol consumption | - | - | - |
| Obesity (n=977) | 0.95 | 0.63 | 1.45 |
| Unhealthy lifestyle index (n=977) | 1.01 | 0.69 | 1.47 |
| Undetected hypertension (n=743) | 0.63 | 0.31 | 1.54 |
| Undetected diabetes (n=601) | 0.69 | 0.31 | 17.76 |
| Undetected chronic obstructive pulmonary disease (n=108) | 4.37 | 1.07 | 2.41 |
| Uncontrolled hypertension (n=605) | 1.42 | 0.83 | 1.27 |
| Uncontrolled diabetes (n=506) | 0.67 | 0.36 | 1.49 |
| Uncontrolled chronic obstructive pulmonary disease | - | - | - |

*A Depression, Anxiety, Stress Scale-21 score of ≥14 indicated having depressive symptoms. Mixed ordinal logistic regression models quantified the association between depressive symptoms and lifestyle index. The associations between depressive symptoms and all other outcomes were quantified with mixed logistic regression models. All models included municipality as a random effect and were adjusted for age, sex, work status, education level, living in a rural or urban setting, and ethnicity except only adjustments for age and sex were included in the model of the association between depression and undetected chronic obstructive pulmonary disease due to few cases. We did not include a regression for the association between depressive symptoms and alcohol consumption because there were no cases of depressed people drinking alcohol in the last 30 days, therefore depressive symptoms predicted the outcome perfectly. We also did not include a regression model for the association between depressive symptoms and uncontrolled COPD because there were too few cases. Subsamples for undetected hypertension, diabetes and COPD included all participants with a self-reported physician diagnosis or pathological findings for the given disease (systolic blood pressure ≥ 140mmHg or diastolic blood pressure ≥ 90mmHg for hypertension; HbA1c ≥ 6.5% for diabetes; PEF < 80% Predicted with breathlessness for six months or longer or cough for at least 3 months for COPD). Subsamples for uncontrolled disease included all participants with a self-reported physician diagnosis for the given disease. The vertical red line indicates the limit of the odds ratio of one.*
