# Supplementary material for: Strengthening Primary Healthcare in Kosovo Requires Tailoring Primary, Secondary and Tertiary Prevention Interventions and Consideration of Mental Health
Source: Front Public Health. 2022 Apr 5;10:794309. doi: 10.3389/fpubh.2022.794309 (PMC9037373; doi:10.3389/fpubh.2022.794309)
Supplement: Supplementary file 2 [file Table_2.DOCX]

Table S2. Association between depressive symptoms (continuous score) and non-communicable disease risk factors (smoking, physical, inactivity, poor nutrition, alcohol consumption, obesity, and unhealthy lifestyle index), undetected and uncontrolled hypertension, diabetes and COPD (Kosovo Non-Communicable Disease Cohort, Kosovo, 2019)

| Outcome | Odds Ratio per one-point increase of depressive symptoms | 95 % confidence interval | |
| --- | --- | --- | --- |
| Currently smoking (n=977) | 1.00 | 0.97 | 1.02 |
| Physical Inactivity (n=977) | 1.02* | 1.00 | 1.05 |
| Poor nutrition (n=977) | 1.00 | 0.97 | 1.02 |
| alcohol (n=977) | 0.90 | 0.81 | 1.01 |
| Obesity (n=977) | 1.00 | 0.98 | 1.02 |
| lifestyl index (n=977) | 1.01 | 0.99 | 1.03 |
| undetected hypertension (n=743) | 0.98 | 0.95 | 1.01 |
| undetected diabetes (n=601) | 0.95* | 0.91 | 1.00 |
| undetected chronic obstructive pulmonary disease (n=108) | 1.07* | 1.00 | 1.15 |
| uncontrolled hypertension (n=605) | 1.01 | 0.98 | 1.04 |
| uncontrolled diabetes (n=506) | 1.00 | 0.97 | 1.03 |
| uncontrolled chronic obstructive pulmonary disease (n=59) | - | - | - |

*Depressive symptoms were assessed using the Depression, Anxiety, Stress Scale-21. Mixed ordinal logistic regression models quantified the association between depressive symptoms and lifestyle index. The associations between depressive symptoms and all other outcomes were quantified with mixed logistic regression models. All models included municipality as a random effect and were adjusted for age, sex, work status, education level, living in a rural or urban setting, and ethnicity with exception of alcohol, which was reduced to adjustment for only age, sex and ethnicity. Subsamples for undetected hypertension, diabetes and COPD included all participants with a self-reported physician diagnosis or pathological findings for the given disease (systolic blood pressure ≥ 140mmHg or diastolic blood pressure ≥ 90mmHg for hypertension; HbA1c ≥ 6.5% for diabetes; PEF < 80% Predicted with breathlessness for six months or longer or cough for at least 3 months for COPD). Subsamples for uncontrolled disease included all participants with a self-reported physician diagnosis for the given disease. The vertical red line indicates the limit of the odds ratio of one.*
